# The Microplastic–PFAS Nexus: From Co-Occurrence to Combined Toxicity in Aquatic Environments

**DOI:** 10.3390/toxics13121041

**Published:** 2025-11-30

**Authors:** Ping Wang, Yu-Zhen Shi, Qingqing Guan

**Affiliations:** 1 Faculty of Environmental Science and Engineering, Kunming University of Science and Technology, Kunming 650500, China; wangping202506@163.com (P.W.);; 2 School of Chemical Engineering, Xinjiang University, Urumqi 830000, China

**Keywords:** microplastics, per- and polyfluoroalkyl substances (PFAS), combined toxicity, adsorption, trophic transfer, environmental fate, risk assessment, synergistic effects, emerging contaminants, remediation

## Abstract

The pervasive environmental contamination by microplastics (MPs) and per- and polyfluoroalkyl substances (PFAS) represents a critical challenge of the Anthropocene. While historically studied in isolation, a growing body of evidence confirms that these pollutants interact to form a complex and dynamic MP-PFAS Nexus. This review synthesizes current knowledge to elucidate the multifaceted mechanisms of this interaction, where MPs act as vectors, concentrators, and secondary sources for PFAS. We detail how environmental aging and water chemistry modulate adsorption and transport, fundamentally altering the fate of both contaminants. Crucially, the review consolidates evidence demonstrating that co-exposure often leads to synergistic toxicity, disrupting physiological processes from photosynthesis in algae to lipid metabolism and neurogenesis in animals, with significant implications for trophic transfer. The nexus also presents formidable challenges for water treatment and soil remediation, while simultaneously offering opportunities for targeted destructive technologies like pyrolysis. Furthermore, we explore the emerging threats of this complex to human health via seafood and water, and the amplifying feedback of climate change. Finally, we argue that current regulatory frameworks, which assess pollutants individually, are inadequate and must evolve to account for combined effects. This review underscores the imperative to reframe MPs and PFAS as an interconnected pollutant system, necessitating integrated research and policy for effective environmental risk assessment and management.

## 1. Introduction

The Anthropocene is characterized by the widespread dissemination of anthropogenic chemicals, among which microplastics (MPs) and per- and polyfluoroalkyl substances (PFAS) represent two of the most pervasive and persistent classes of environmental contaminants. MPs, synthetic polymer particles smaller than 5 mm, are ubiquitous in global ecosystems, from the deepest marine trenches to remote alpine lakes, a consequence of the massive production and inadequate disposal of plastic waste [1,2]. Their persistence stems from their synthetic, recalcitrant chemical structures, while their environmental impact is exacerbated by their small size, high surface area-to-volume ratio, and capacity to act as vectors for other pollutants. Similarly, PFAS, a family of thousands of synthetic organofluorine compounds, have earned the moniker forever chemicals due to the exceptional strength of their carbon-fluorine bonds, rendering them highly resistant to environmental degradation [3,4,5]. Renowned for their water- and grease-repellent properties, PFAS have been used extensively in industrial and consumer products, leading to their global detection in water, soil, air, and biota. The environmental legacy of both MPs and PFAS is defined by their durability, bioaccumulative potential, and the profound, long-term threats they pose to ecological security and human health.

While the individual environmental behaviors and toxicologies of MPs and PFAS have been extensively researched, a critical paradigm shift is emerging: these pollutants rarely exist in isolation. A growing body of field evidence confirms that their co-occurrence is the rule, not the exception, across diverse environmental compartments. Studies have documented their simultaneous presence and significant correlations in wastewater treatment plants, where MPs were identified as direct carriers of PFAS such as perfluorooctanoic acid (PFOA) and perfluorobutanoic acid (PFBA) [6]. In freshwater systems like Taihu Lake, China, the distribution of specific PFAS, including the alternative compound HFPO-DA (GenX), was directly linked to the concentration and size of co-occurring MPs [7]. This co-contamination extends to riverine and estuarine systems, with road dust identified as a potential source for both contaminants in stormwater runoff, ultimately discharging them into open water bodies [8,9]. Even the terrestrial environment and the Arctic are not spared, with PFAS, MPs, and other plastic-associated additives being detected as co-contaminants in agricultural soils and seabirds, suggesting complex transport pathways and interactions [10,11]. This ubiquitous co-occurrence suggests that their individual risk assessments provide an incomplete and potentially underestimated picture of their true environmental impact.

**Figure 1:**
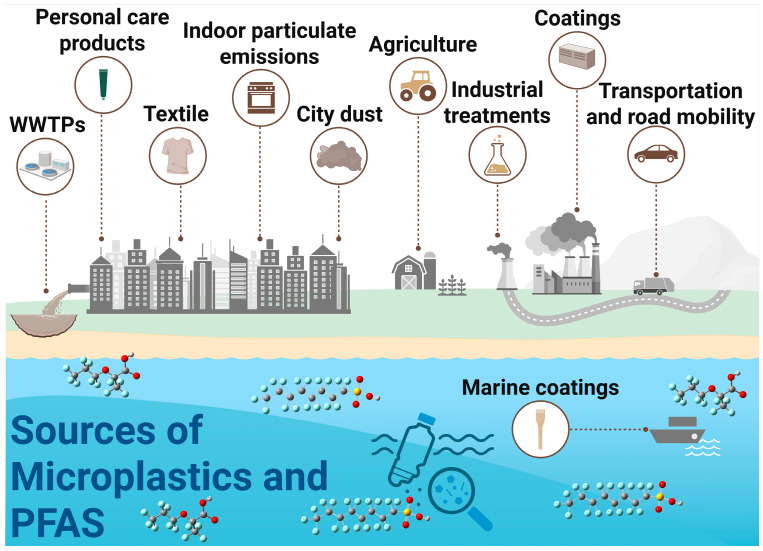
Sources of PFAS and microplastics leading to their mixed contamination of aquatic ecosystems.

This review introduces and explores the concept of the MP-PFAS Nexus to describe the dynamic and multifaceted interactions between these two pollutant classes. This framework moves beyond the simplistic view of MPs as passive vectors to encompass a system where the physical, chemical, and biological interactions between MPs and PFAS alter the environmental fate, transport, bioavailability, and ultimate toxicity of both. Foundational field studies have laid the groundwork for this concept, demonstrating that the adsorption of PFAS onto MPs is not a mere laboratory curiosity but an environmentally relevant process [6,7,12]. The nexus is dynamic; factors such as MP aging, biofilm formation, and water chemistry can modulate the strength of these interactions, turning MPs into both sinks and secondary sources of PFAS (Figure 1) [12,13,14]. Critically, emerging evidence indicates that the combined effects of this nexus on aquatic organisms are often synergistic, leading to enhanced oxidative stress, developmental failures, and metabolic disruptions that would not be predicted from the toxicity of each contaminant alone [8,15,16,17]. Therefore, understanding this synergistic relationship is paramount for accurate ecological risk assessment.

The primary objectives of this review are: To synthesize the current understanding of the interaction mechanisms between MPs and PFAS, focusing on the roles of MP polymer type, aging, and environmental conditions. To critically evaluate the evidence demonstrating how the MP-PFAS nexus alters the environmental fate, transport, and trophic transfer of these pollutants. To consolidate findings on the combined toxicological effects of MP and PFAS co-exposure across different levels of biological organization, from molecular to ecosystem-level responses and to identify critical knowledge gaps and propose future research directions for better characterizing the risks and developing mitigation strategies targeted at this complex pollutant system. By reframing MPs and PFAS as an interconnected nexus, this review aims to catalyze a more holistic and accurate approach to environmental monitoring, risk assessment, and remediation of these persistent pollutants.

## 2. Mechanisms of Interaction: Beyond Simple Adsorption

The foundational step in the formation of the MP-PFAS nexus is the initial adsorption of PFAS onto the plastic surface. However, this process is far from a simple, uniform phenomenon. It is a complex interplay governed by the physicochemical properties of both the microplastic and the PFAS molecule, as well as the transformative effects of environmental weathering. Understanding these mechanisms is crucial to predicting the behavior, mobility, and ultimate bioavailability of this combined pollutant complex.

### 2.1. MPs as Vectors and Concentrators

**Figure 2:**
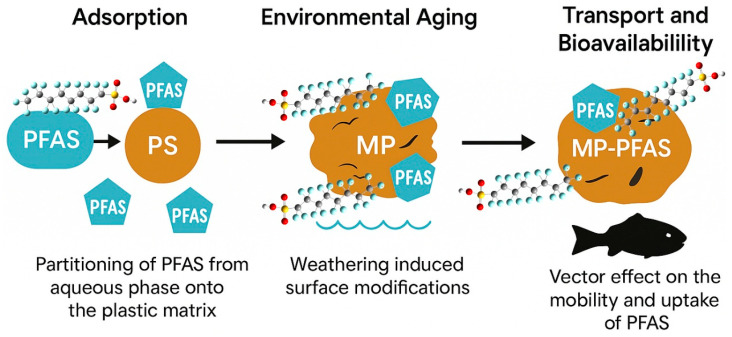
Interaction of microplastics and PFAS where MPs are acting as a vector for PFAS transport.

The role of microplastics (MPs) as vectors for per- and polyfluoroalkyl substances (PFAS) transcends the simplistic model of passive sorption (Figure 2). It is a dynamic, multi-stage process governed by a complex interplay of physicochemical forces that transform the environmental fate and biological impact of both pollutants. This vectoring process begins with the critical adsorption phase, where PFAS partition from the aqueous phase onto the plastic matrix. The efficiency of this initial adsorption is not uniform; it is highly dependent on the specific polymer type, the molecular structure of the PFAS, and the transformative effects of environmental weathering. Polystyrene (PS), by virtue of its aromatic backbone, often demonstrates a superior adsorption capacity for PFAS compared to polyolefins like polyethylene (PE), suggesting that specific interactions such as π-π bonding between the electron-rich benzene rings of PS and the electron-deficient fluorocarbon chains of PFAS play a significant role [18]. Furthermore, the hydrophobic effect serves as a primary driver, particularly for long-chain PFAS like PFOA and PFOS. The strongly hydrophobic fluorinated tail of these molecules seeks to minimize its contact with the polar water environment, favoring association with the polymeric surface of MPs. This mechanism is so potent that adsorption can be dramatically amplified in real-world settings. Scott et al. [19] provided compelling evidence for this, finding that the adsorption of PFAS onto field-incubated microplastics was 24 to 259 times greater than what was observed on pristine plastics in controlled laboratory experiments. This stark discrepancy underscores the critical importance of the environmentally acquired coating, or the eco-corona, which will be elaborated upon later.

The intrinsic properties of the PFAS molecules themselves are equally critical in determining their affinity for microplastic surfaces. A consistent structure-activity relationship has been observed whereby the chain length of the PFAS directly correlates with its adsorption potential. Brahana et al. [20] systematically demonstrated this by investigating the uptake of a series of perfluoroalkyl carboxylic acids (PFCAs) onto polyethylene, concluding that as the number of carbon atoms in the fluoroalkyl chain increases, there is a corresponding increase in the adsorption of PFCAs onto microplastics. This is a direct consequence of the increasing hydrophobicity and molecular volume of longer-chain compounds, which enhances their driving force to escape the aqueous phase. However, the story is further complicated by the impact of environmental aging. When MPs are exposed to UV radiation, mechanical abrasion, and oxidative processes, their surface chemistry is fundamentally altered. As Ning et al. [13] documented, aged MPs exhibit an increased carbonyl index, indicating the formation of oxygen-containing functional groups such as hydroxyl and carboxyl groups. This weathering process increases surface roughness and can create new binding sites, thereby modifying the MP’s electrostatic properties and overall capacity to concentrate pollutants, making aged MPs more potent vectors than their virgin counterparts.

The ultimate environmental consequence of this complex adsorption process is the vector effect, wherein MPs alter the transport and bioavailability of PFAS. The formation of the MP-PFAS complex can fundamentally change the mobility of both contaminants. Ding et al. [2] reported that while PFAS are typically dissolved and mobile, their association with dense MPs could lead to enhanced deposition and accumulation in sediments. Conversely, sorption to buoyant, small MPs could facilitate the long-range transport of PFAS to pristine environments. Most critically, this association dramatically enhances bioavailability and trophic transfer. Filter-feeding and sediment-ingesting organisms constantly consume MPs. When these particles are loaded with PFAS, they act as a Trojan horse, delivering a concentrated dose of contaminants directly into the organism’s digestive system. The unique conditions of the gut, including surfactants, digestive enzymes, and a sharp pH gradient, can promote the desorption of PFAS from the MP surface, leading to internal exposure levels far exceeding those from waterborne contamination alone. This mechanism has been demonstrated to increase the assimilation efficiency of persistent pollutants in aquatic food webs [21], confirming that the MP-PFAS nexus is not merely a chemical phenomenon but a profound biological threat.

### 2.2. The Aging Factor: Transforming Microplastic Vector Capacity

The journey of microplastic through the environment is one of constant physical and chemical transformation, and these aging processes profoundly redefine its role in the PFAS nexus. A pristine plastic item shed from a consumer product bears little resemblance to the particle that eventually interacts with PFAS in a lake or ocean. Aging through ultraviolet (UV) radiation, mechanical abrasion, and biological fouling fundamentally alters the microplastic’s surface chemistry, morphology, and thus, its affinity for different PFAS compounds. The most significant chemical change induced by UV exposure is photo-oxidation, which breaks polymer chains and introduces oxygen-containing functional groups such as hydroxyl (-OH), carbonyl (C=O), and carboxyl (-COOH) onto the plastic surface. Ning et al. [13] directly quantified this change during their investigation of PFAS adsorption, reporting an increase in the carbonyl index of MPs after treatment with UV irradiation and persulfate oxidation. This oxidation creates a more hydrophilic and polar surface, which can initially seem counterintuitive for the adsorption of hydrophobic pollutants. However, while long-chain PFAS adsorption remains largely governed by hydrophobic interactions, these newly formed oxygenated groups can participate in specific interactions, such as hydrogen bonding with the functional head groups of PFAS (e.g., the carboxylic acid of PFOA), and increase the overall surface reactivity and sorption capacity of the aged particle.

The physical manifestation of aging is equally critical. Mechanical abrasion from wave action, sediment scouring, and wind erosion progressively fragments MPs into smaller particles and creates a roughened, pitted surface topography. This physical weathering does not occur in isolation; it works synergistically with chemical degradation. UV radiation embrittles the polymer, making it more susceptible to mechanical breakdown. The resulting increase in surface area and the creation of micro-crevices provide a greater number of potential sites for PFAS to adsorb and become trapped. This combination of increased specific surface area and oxidative functionalization means that an aged MP can often act as a far more potent concentrator for a wider range of PFAS than a smooth, pristine particle. The study by Shi et al. [22] on the adsorption of PFAS and microcystins onto weathered microplastics supports this, observing that weathering introduced hydrophilic oxygen-containing groups that altered adsorption behavior, with virgin plastics sometimes exhibiting higher adsorption for certain contaminants due to their dominant hydrophobic character, highlighting the nuanced and pollutant-specific outcomes of the aging process.

Perhaps the most biologically significant aging process is the development of a biofilm, or an eco-corona. Within hours to days of entering an aquatic environment, MPs become colonized by a complex layer of microorganisms, algae, and a matrix of extracellular polymeric substances (EPS). This biofilm fundamentally rebrands the MP, transforming it from a synthetic material into a biologically active particle. Liu et al. [7], in their field investigation of a large shallow lake, revealed that this biofilm is not a passive spectator but an active accelerator of the MP-PFAS nexus. They found that the biofilm on the surface of MPs significantly accelerates accumulation process of PFAS, particularly for the alternative compound HFPO-DA. The biofilm matrix, rich in proteins, polysaccharides, and other organic molecules, provides a dense, gelatinous medium with immense sorption capacity. It can concentrate PFAS from the water column through various mechanisms, effectively pre-loading the MP before the contaminants even reach the native polymer surface. This makes the biofilm-coated MP a highly efficient, mobile micro-habitat for PFAS accumulation. Furthermore, the biofilm can facilitate the irreversible adsorption of PFAS, promoting high cumulative flux into sediments and enhancing the potential for trophic transfer, as the entire complex, plastic, biofilm, and contaminant, becomes a palatable and toxic food source for a range of aquatic organisms. In summary, aging does not deactivate MPs; it equips them with new chemical functionalities and biological partnerships that dramatically enhance their capacity to vector PFAS in the environment.

### 2.3. MPs as a Secondary Source: Leaching and Legacy Contamination

Beyond their role as concentrators and transport vehicles, a growing body of evidence positions microplastics as a direct and significant secondary source of PFAS contamination in aquatic environments. This paradigm shift acknowledges that MPs are not merely clean surfaces that passively adsorb pollutants; rather, they can be inherently loaded with PFAS from their production lifecycle or through subsequent contamination, and these substances can be leached back into the environment. The presence of PFAS in commercial plastic products stems from two primary pathways: their intentional use as polymer processing aids during manufacturing, particularly in fluorinated polymers, and their non-intentional incorporation through adsorption from contaminated water or air during use or disposal. This intrinsic contamination was starkly highlighted by Ateia et al. [23], who raised the critical question, Should we continue using pure polymers as surrogates for real MPs? Their systematic study, which compared the sorption of micropollutants on a large set of well-characterized real-world MPs versus pure polymers, found that real MPs often had higher normalized uptake. This was attributed to the presence of industrial additives and fillers (e.g., talc, glass fiber), which create a more complex and reactive surface chemistry. More importantly, their work suggested that the complex composition of real-world plastics could facilitate a greater and more diverse interaction with contaminants, implying that the plastics themselves are a reservoir of chemical constituents.

Compelling empirical evidence for this leaching phenomenon was provided by Ma et al. [6] in their comprehensive study of a wastewater treatment plant (WWTP). They not only confirmed the concurrent presence of MPs and PFAS but also identified significant correlations between them, suggesting shared sources. Crucially, their laboratory verification experiments demonstrated that PFAS could indeed be leached from the MPs themselves in aqueous environments. They reported that commercial MPs exhibited a high leaching potential, with the combined concentration of PFOS, PFOA, and PFBA reaching up to 10.4 ng/mL in the leaching solution. A particularly revealing finding was that in sorption/desorption experiments, PFOS demonstrated a desorption efficiency exceeding 120%. This counterintuitive result, where more PFAS was released than was initially adsorbed in the experiment, serves as definitive proof that a significant reservoir of PFAS was inherent to the MPs themselves, being released from the polymer matrix. This transforms our understanding of the MP-PFAS nexus: MPs are not just sinks but also active sources, continually releasing these forever chemicals into the water.

The environmental implications of this secondary source role are profound [17,24,25,26]. It means that the millions of tons of plastic pollution in our oceans and freshwater systems represent a continuous, diffuse, and long-term source of PFAS contamination, independent of direct discharges from industrial or firefighting activities. The leaching process can be influenced by environmental conditions such as pH, temperature, and the presence of organic solvents or surfactants, which can accelerate the diffusion of PFAS out of the polymer matrix. This is especially concerning for biodegradable plastics, which are designed to fragment and degrade, potentially releasing their chemical payload more rapidly. Therefore, the role of MPs in the PFAS cycle is dualistic: they act as both a sponge that concentrates PFAS from the environment and a time-release capsule that introduces legacy PFAS into new environments. This necessitates a reevaluation of global PFAS budgets and remediation strategies, as managing point sources alone will be insufficient without addressing the vast and mobile reservoir of PFAS-laden plastic pollution [27].

### 2.4. PFAS and MPs Type: A Compound Specific Perspective

For perfluoroalkyl acids (PFAAs) like PFOA, PFOS, and their alternatives, the primary driver of adsorption is the hydrophobic effect. The strength of this interaction is directly correlated with the length of the fluorinated carbon chain. Long-chain PFAAs (e.g., PFOA, PFOS, PFNA) possess a highly hydrophobic fluorinated tail that strongly partitions away from the aqueous phase onto the plastic polymer. This is quantitatively demonstrated by Brahana et al. [20], who found a direct correlation between PFCA chain length and adsorption to polyethylene. In contrast, short-chain PFAAs (e.g., PFBA, PFBS) and some alternatives like GenX (HFPO-DA), with their shorter chains or ether oxygen groups, are more hydrophilic. Their adsorption is weaker and more readily influenced by secondary mechanisms, such as electrostatic interactions with charged functional groups on weathered MPs [14]. Consequently, long-chain PFAAs generally exhibit higher sorption coefficients (Kd) on MPs, making them more likely to be sequestered and transported, whereas short-chain compounds remain more freely dissolved and mobile in the water column.

The behavior of polyfluoroalkyl substances (e.g., fluorotelomer alcohols (FTOHs), perfluorooctane sulfonamide (FOSA)) is more complex and less studied. These precursors are typically more hydrophobic than their terminal PFAAs due to their non-fluorinated segments. This suggests they could have an even higher intrinsic affinity for MPs. Meng et al. [18] observed higher adsorption of FOSA compared to PFOA on various polymers. This strong initial sorption is critical because MPs can then become a mobile reservoir for these precursors, which may subsequently transform into more persistent PFAAs. The unique microenvironment of an MP’s surface, especially when coated with a biofilm, could potentially catalyze this transformation, turning the MP into a travelling reactor that gradually releases more toxic and persistent end-products into the environment.

These compound-specific interactions are further modulated by the MP’s properties. Aromatic polymers like polystyrene (PS) can engage in specific π-π interactions with the fluorocarbon chain, leading to higher adsorption capacities for certain PFAS compared to polyolefins like polyethylene (PE) [18]. Furthermore, environmental aging introduces oxygen-containing functional groups that can create new binding sites via hydrogen bonding or alter electrostatic interactions. This weathering can differentially affect PFAS classes: it may increase the adsorption of ionizable short-chain PFAAs through polar interactions, while potentially having a lesser effect on the adsorption of long-chain PFAAs, which is already dominated by strong hydrophobic forces [13].

### 2.5. Influence of Water Chemistry: Modulating the MP-PFAS Complex

The stability and strength of the microplastic–PFAS complex are not intrinsic properties but are dynamically governed by the chemistry of the surrounding aqueous environment. Key parameters, pH, salinity, and dissolved organic matter (DOM), act as master variables, capable of either strengthening the bond between pollutant and plastic or decisively pulling them apart, thereby dictating the environmental mobility and bioavailability of the entire complex.

The pH of the water exerts a profound influence by modulating the surface charge of both the microplastic and the PFAS molecule. In neutral to alkaline conditions typical of many natural waters, many PFAS, such as perfluorooctanoic acid (PFOA) and perfluorooctanesulfonic acid (PFOS), exist in their anionic, deprotonated forms. Concurrently, environmentally weathered MPs, adorned with oxygen-containing functional groups (e.g., carboxylates), also carry a negative surface charge. This creates a significant electrostatic repulsion that can inhibit the close approach of the PFAS to the MP surface. As Salawu et al. [14] demonstrated in their mechanistic study, increased pH increased electrostatic repulsion, which negated PFAS adsorption. Conversely, under more acidic conditions, the MP surface may become less negative through protonation, and the functional groups of some PFAS may also protonate, reducing this repulsive barrier and allowing the stronger, attractive hydrophobic interactions to dominate, thereby enhancing adsorption.

Salinity, or ionic strength, plays a similarly critical but often opposing role. The presence of dissolved ions, particularly divalent cations like calcium (Ca^2+^) and magnesium (Mg^2+^) found in seawater and hard freshwater, can effectively screen the negative charges on both the MP and the anionic PFAS. This charge screening reduces the electrostatic repulsion, thereby facilitating closer contact and stronger adsorption. Salawu et al. [14] confirmed this, noting that higher ionic strength favored PFAS adsorption by decreasing electrostatic repulsion. In essence, salts can act as a bridge or a catalyst for the formation of the MP-PFAS complex. This has significant implications for estuarine and marine environments, suggesting that the transition from freshwater to saltwater can trigger increased scavenging of PFAS from the water column onto the abundant marine microplastic debris.

Perhaps the most complex modulator is dissolved organic matter (DOM), a heterogeneous mixture of organic compounds ubiquitous in natural waters. DOM competes directly with PFAS for sorption sites on the microplastic surface. The humic and fulvic acids within DOM can bind strongly to the weathered, functionalized surfaces of MPs, effectively blocking potential binding sites and reducing the overall adsorption capacity for PFAS. Furthermore, DOM can impose an electrosteric hindrance; the large, complex molecules of DOM adsorbed onto an MP create a physical and repulsive barrier that prevents PFAS from reaching the sorption sites. As a result, the presence of DOM generally leads to a decrease in PFAS adsorption onto MPs, maintaining a higher concentration of mobile, bioavailable PFAS in the dissolved phase. However, a more intricate dynamic can also occur, where PFAS may co-sorb with DOM, forming a ternary complex. This is why Ateia et al. [23] crucially noted that the evaluation of MPs sorption behavior without NOM preloading can result in a significant underestimation for their actual values, emphasizing that laboratory studies using pure water vastly oversimplify the real-world scenario.

While pH, ionic strength, and dissolved organic matter (DOM) are established as master variables controlling the MP-PFAS interaction, several other critical environmental parameters can significantly alter adsorption dynamics and the stability of the complex. A more holistic view must incorporate the roles of temperature, the presence of co-contaminants, and the intrinsic properties of the aqueous matrix itself. Temperature is a fundamental driver of adsorption thermodynamics and kinetics. An increase in temperature typically enhances the diffusion rate of PFAS molecules, potentially accelerating the attainment of sorption equilibrium. However, its effect on sorption capacity is complex and system-dependent. Adsorption is an exothermic process for many hydrophobic interactions; therefore, higher temperatures can decrease the sorption capacity of long-chain PFAAs onto MPs, as described by the van’t Hoff equation. In contrast, for interactions driven by endothermic processes, such as the pore diffusion of larger molecules or specific chemical interactions, sorption may increase with temperature. Salawu et al. [14] provided evidence for the spontaneity of PFAS adsorption onto secondary MPs, but the specific influence of temperature on this process remains an area requiring systematic investigation, particularly under field-relevant fluctuating thermal regimes.

**Figure 3:**
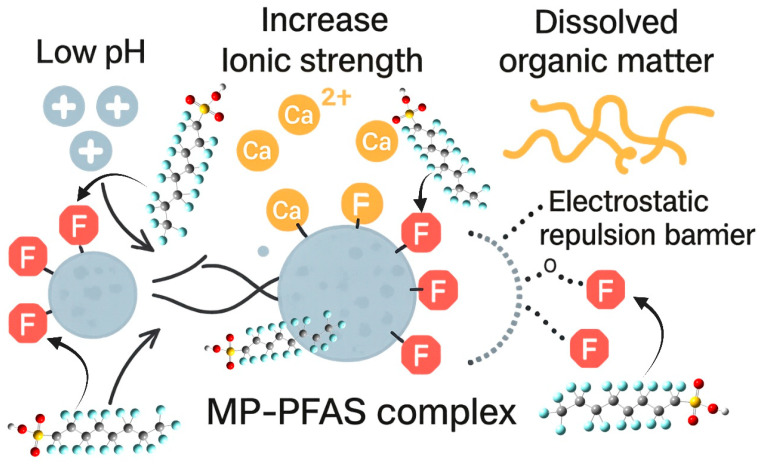
Impact of water chemistry (pH, ionic strength, and dissolved organic matter) on the sorption behavior of PFAS and MPs.

The aquatic environment is a cocktail of pollutants, and the presence of co-contaminants can lead to competitive or synergistic effects on PFAS adsorption. Heavy metals can act as cationic bridges, similar to Ca^2+^ and Mg^2+^, enhancing the adsorption of anionic PFAS. Conversely, other organic surfactants or non-PFAS industrial chemicals can compete directly with PFAS for limited sorption sites on the MP surface (Figure 3). This competition can be especially significant for weathered, high-surface-area MPs where the sorption capacity, while enhanced, is still finite. The study by Ateia et al. [23] on the sorption of multiple micropollutants on a large set of real-world MPs underscores this complexity, revealing that the presence of other contaminants can significantly alter the predicted partitioning behaviour, leading to an underestimation or overestimation of any single chemical’s affinity. Furthermore, the overall water chemistry and biological activity create a dynamic system. The divalent-to-monovalent cation ratio is more significant than ionic strength alone, as divalent cations are far more effective in screening negative charges. In high-hardness water, the MP-PFAS complex is likely to be more stable. Moreover, the formation of a biofilm (eco-corona), as discussed in Section 2.3, fundamentally rebrands the MP surface. This living layer is not static; its composition and charge change over time and can actively metabolize or transform sorbed contaminants. Liu et al. [7] showed that biofilms accelerate PFAS accumulation on MPs, but they may also facilitate the biodegradation of precursor PFAS, altering the chemical profile of the contaminants associated with the plastic.

### 2.6. The Expanding Universe of PFAS: Beyond PFAAs to Precursors and Alternatives

While this review frequently cites studies on perfluoroalkyl acids (PFAAs) like PFOA, PFOS, and their short-chain alternatives (e.g., PFBA, PFBS, GenX), it is crucial to acknowledge that the term ‘PFAS’ encompasses a vast and complex family of over 12,000 synthetic compounds. This includes a critical sub-class: polyfluoroalkyl substances, also known as precursor compounds. These precursors, such as fluorotelomer alcohols (FTOHs), perfluoroalkyl sulfonamides (e.g., FOSA), and polyfluoroalkyl phosphate esters (PAPs), contain at least one carbon atom that is not fully fluorinated. They are themselves contaminants of concern and can undergo environmental transformation, both abiotically and biotically, into terminal PFAAs like PFOA and PFOS [18]. The focus on PFAAs in many mechanistic studies, including those cited herein, is often due to their known persistence, bioaccumulation potential, and the relative availability of analytical standards. However, the behavior of precursors within the MP-PFAS nexus may be distinctly different. Their typically higher hydrophobicity and different chemical functionalities could lead to stronger sorption to MPs, and their subsequent transformation could be catalyzed by the unique microenvironment of the MP surface or its associated biofilm, making MPs not just vectors but also potential reactors for PFAS transformation. While GenX (HFPO-DA) is a prominent PFOA alternative, other compounds like F-53B (a chlorinated polyfluoroether sulfonate used in the Chinese electroplating industry) are of rising global concern. Recent studies have shown that F-53B can exhibit even stronger adsorption to certain MPs and induce greater toxicological effects in co-exposure scenarios compared to legacy PFOS [28]. The limited discussion on the full spectrum of polyfluoroalkyl substances and diverse alternatives represents a significant knowledge gap. Future research must prioritize investigating these understudied compounds to fully understand the scope and risk of the MP-PFAS nexus.

## 3. Environmental Fate and Transport: A Coupled Journey

**Figure 4:**
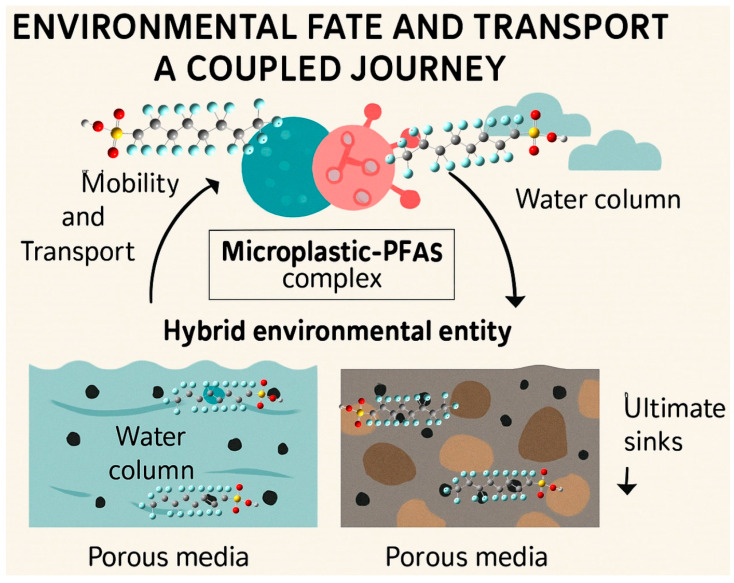
Environmental fate and transport of PFAS and MPs as coupled journal from water, soil, and air leading to the ultimate sink of mixed contaminants.

The formation of the microplastic–PFAS complex creates a new, hybrid environmental entity whose behavior is distinct from that of either contaminant in isolation (Figure 4). This coupling fundamentally alters the mobility and transport pathways of both pollutants in porous media (like sediments and soils) and water columns, with consequences for their distribution, long-range transport, and ultimate sinks.

### 3.1. Pollution Sources and Convergent Transport Pathways to Aquatic Systems

The ubiquity of the MP-PFAS nexus in aquatic environments is a direct consequence of multiple, often overlapping, pollution sources and their subsequent transport pathways. Understanding these origins is critical for effective source control and remediation. The major sources and their convergent pathways, as illustrated in Figure 1, can be categorized as urban and industrial runoff, WWTPs, agriculture and land application, atmospheric deposition and plastic degradation. This is a primary pathway where MPs and PFAS actively interact. Urban areas contribute MPs from tire wear, synthetic textile fibers, and fragmented plastic litter. Concurrently, they are a source of PFAS from historical use of aqueous film-forming foams (AFFFs) at fire-training facilities and leaching from weatherproof coatings and consumer products. Stormwater and surface runoff then transport both contaminants simultaneously. During this transport, MPs can adsorb PFAS from the runoff water, forming the initial MP-PFAS complex before even reaching a major water body. This is supported by Pramanik et al. [9], who identified road dust as a potential origin of both MPs and PFAS in stormwater.

WWTPs are significant convergence points. They receive a complex influent containing MP fibers from laundry and PFAS from industrial discharge, domestic products (e.g., cosmetics, cleaning agents), and landfill leachate. As demonstrated by Ma et al. [18], significant interactions occur within the WWTP itself, with MPs acting as both carriers and sources of PFAS. While some of the complex is removed with the sludge (biosolids), a substantial fraction is discharged with the treated effluent, making WWTPs a continuous point source of the pre-formed MP-PFAS nexus into rivers and lakes. The practice of applying PFAS- and MP-contaminated biosolids as fertilizer to agricultural land creates a significant diffuse source. This introduces both contaminants directly to the soil. Subsequent irrigation and rainfall events can then cause runoff that transports soil-bound MPs and their sorbed PFAS into adjacent waterways. This pathway directly links human waste management and agricultural practices to the contamination of aquatic ecosystems.

An often-overlooked pathway is atmospheric transport. Volatile PFAS precursors (e.g., FTOHs) and lightweight MPs can be transported long distances through the air. They are subsequently deposited via rainfall or dry deposition into aquatic systems. Furthermore, the ongoing degradation of larger plastic debris in the environment, from fishing gear to packaging waste, acts as a continuous in situ source of secondary MPs, which can immediately interact with dissolved PFAS in the water column, continually refreshing the pool of available vectors. These pathways are not isolated; they are interconnected. Atmospheric deposition can contaminate agricultural land, which then contributes to runoff. This multi-source, multi-pathway reality underscores why the MP-PFAS nexus is so pervasive and resilient, necessitating integrated management strategies that target these key sources to disrupt the cycle of contamination.

### 3.2. Altered Mobility: Mixed MPs Impact on PFAS Transport

In porous media, the MP-PFAS complex can exhibit transport behavior that is a composite of its components. PFAS, particularly the shorter-chain, more hydrophilic varieties, are typically highly mobile in groundwater and saturated soils due to their solubility and anionic repulsion from negatively charged soil particles. However, when sorbed onto microplastics, their mobility becomes tied to the fate of the particle. If the MP carrier is itself mobile, a small, buoyant, and low-density polyethylene particle, it can facilitate the transport of PFAS, acting as a vehicle that enables the contaminants to penetrate deeper into aquifers or travel further than they would alone. This is a significant concern for groundwater contamination plumes emanating from landfills or contaminated sites where both pollutants co-occur. Conversely, if the MP is large, dense, or readily filtered by the soil matrix, the complex can experience enhanced retention. The PFAS, which would otherwise be mobile, is effectively pulled out of the water phase by the deposition of its MP carrier, leading to an accumulation of PFAS in the sediment or soil layers. Zhao et al. [29] demonstrated this principle, showing that the transport of polytetrafluoroethylene (PTFE) MPs in porous media was influenced by PFAS co-transport, with the recovery rate of PTFE decreasing under conditions that favor deposition (lower pH, higher ionic strength), thereby also sequestering the associated PFAS.

In the water column, the coupling similarly dictates the contaminants’ trajectory. The complex’s buoyancy, density, and size determine whether it remains suspended, is transported over long distances by currents, or settles out to become a constituent of benthic sediments. The role of the biofilm, as identified by Liu et al. [7], is critical here. The biofilm not only enhances PFAS accumulation but also significantly increases the density and effective size of the MP. This biofouling can cause even buoyant plastics to lose their buoyancy over time, a process known as biological sinking. As Liu et al. [7] noted, the biofilm on the surface of MPs significantly accelerates this accumulation process, referring to the high cumulative flux of HFPO-DA into sediments. This means that the MP-PFAS nexus can actively promote the vertical transport of PFAS from the water column to the sediment, shifting the contamination from a pelagic to a benthic problem. Once in the sediments, these complexes can be re-suspended by bioturbation or storm events or can become a long-term reservoir, continuously releasing PFAS into the porewater and posing a persistent risk to sediment-dwelling organisms.

**Figure 5:**
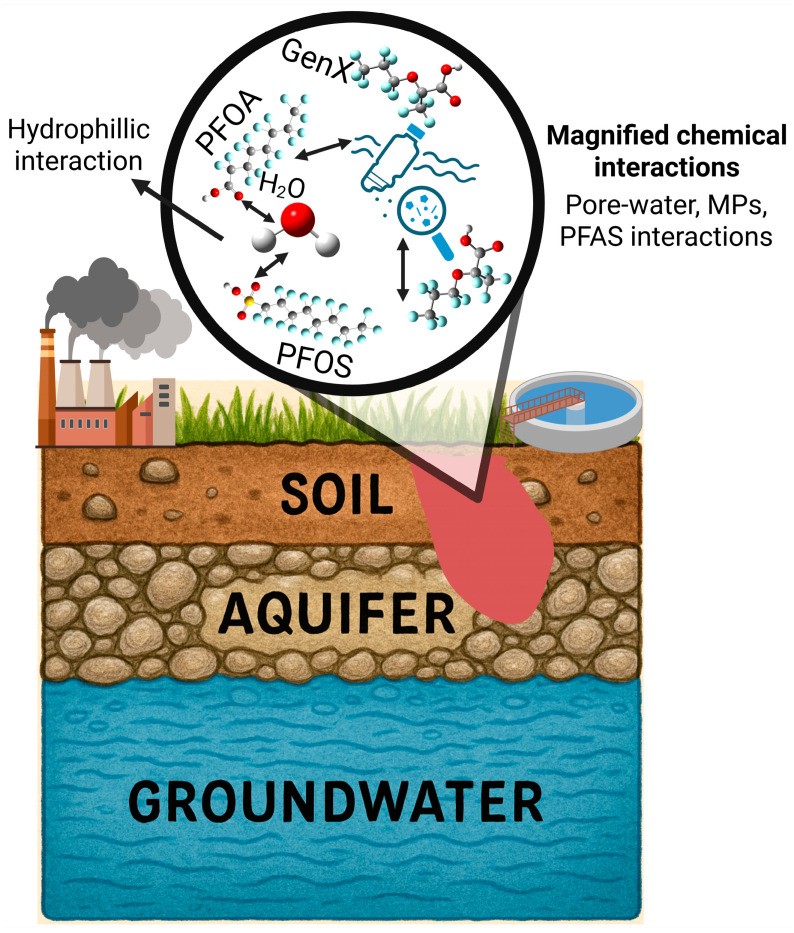
Mechanisms of PFAS and microplastics interaction, thereby influencing the transport and mobility of PFAS and microplastics.

Therefore, the coupled journey of the MP-PFAS complex creates a scenario of divergent and context-dependent mobility. The same nexus that can facilitate the long-range horizontal transport of PFAS in surface waters can also lead to its accelerated vertical deposition in other environments (Figure 5). This dual potential make predicting the environmental distribution of both MPs and PFAS immensely more complex, as their fate is now intertwined and highly sensitive to local conditions such as flow dynamics, sediment type, and biological activity.

### 3.3. Trophic Transfer and Bioaccumulation: The Trojan Horse Effect

The formation of the MP-PFAS complex has profound implications for the entry and movement of these forever chemicals through aquatic food webs. Evidence is mounting that microplastics do not merely add to the background level of PFAS exposure but actively facilitate their uptake and can alter their biomagnification potential, effectively acting as a Trojan Horse that bypasses traditional physiological barriers. The initial entry of the MP-PFAS complex into the food web occurs primarily through filter-feeding and benthic invertebrates. Organisms like *Daphnia magna*, which indiscriminately consume particles in the size range of MPs, are particularly vulnerable. Soltanighias et al. [15] demonstrated that co-exposure to PFAS and MPs caused developmental failures and reduced growth in Daphnia, with combined effects being predominantly additive and synergistic. This indicates that the MP vector introduces a toxic burden that disrupts physiological function at this critical foundation level. The efficiency of this uptake is influenced by the PFAS class; more hydrophobic long-chain PFAAs and precursor compounds likely have a higher potential for MP-mediated uptake in these organisms due to their stronger sorption to the particles. As the nexus moves up the food chain, its consequences become amplified in secondary consumers and predators. A landmark study by Granby et al. [21] on Atlantic salmon provided direct evidence of this. Fish fed a diet with contaminants sorbed to MPs accumulated more persistent organic pollutants and exhibited slower depuration rates than those fed a diet with contaminants alone. Crucially, the study found that MPs caused a greater proportional uptake of higher molecular weight, more hydrophobic congeners. This finding is directly applicable to PFAS, suggesting that the MP vector can shift the internal PFAS profile in fish towards the more persistent and bioaccumulative long-chain PFSAs (e.g., PFOS) and PFCAs (e.g., PFOA, PFNA). This is because these compounds have a higher affinity for both the MP and, subsequently, the lipid-rich tissues of the fish.

The transfer of the MP-PFAS complex is not confined to a single ecosystem. The nexus facilitates the movement of contaminants from freshwater to marine environments and ultimately to human consumers. Yu et al. [30], in their study of marine fish from the South China Sea, found a significant positive correlation between the microplastic burden in fish and the concentration of perfluoroalkyl acids in their tissues. Their structural equation modeling confirmed the contribution of ingested MPs to the body burden of these emerging contaminants. This field-based evidence confirms that the MP-PFAS nexus is active in natural ecosystems and directly contributes to the contamination of seafood. The risk to human health is therefore heightened, as consumption of affected seafood introduces a pre-concentrated dose of both MPs and their associated PFAS payload, with long-chain compounds posing a particular concern due to their biomagnification potential and long human half-lives. The bioaccumulation pathway is further complicated by the distinction between perfluoroalkyl acids (PFAAs) and their polyfluoroalkyl precursors. While PFAAs are the terminal, stable products often measured in tissue, precursor compounds may be the form initially sorbed to MPs. Their strong hydrophobicity suggests efficient MP-mediated uptake. Once ingested by an organism, these precursors can be metabolically transformed into terminal PFAAs. This means that an organism could accumulate significant levels of PFOA or PFOS not from direct exposure, but from the ingestion of MPs carrying their precursors, with biotransformation occurring in vivo. This underscores that assessing only PFAAs in tissue might underestimate the role of MPs, which could be acting as a key vector for the bioaccumulation of the more diverse and less monitored precursor compounds.

The Trojan Horse effect is also compound-specific. The strong sorption of long-chain PFAAs and precursors to MPs makes dietary ingestion a highly efficient exposure route. Once ingested, the gut environment can promote desorption, leading to high assimilation efficiencies, as demonstrated for halogenated contaminants in Atlantic salmon [21]. This can result in a phenomenon where the body burden of long-chain compounds is higher than expected from water exposure alone. For short-chain PFAAs, which are readily absorbed from water and rapidly excreted, the MP-mediated pathway may represent a less significant contribution to the total body burden. However, for precursors, the MP vector could be the primary route of entry, with subsequent in vivo transformation leading to an unexpected accumulation of terminal PFAAs. This underscores that the MP-PFAS nexus can fundamentally alter the bioaccumulation profiles of PFAS in aquatic food webs, favoring the uptake of the more persistent and bioaccumulative long-chain and precursor compounds.

### 3.4. Impact on Remediation and Treatment: Navigating a Complex Nexus

The intimate association between microplastics and PFAS presents a formidable challenge for conventional water and soil remediation technologies, while simultaneously creating opportunities for innovative treatment strategies that target this complex directly. The primary challenge lies in the altered physicochemical behavior of the combined pollutant. In wastewater treatment plants (WWTPs), which are critical barriers for both contaminants, the nexus can compromise treatment efficiency. MPs can shield sorbed PFAS from oxidative destruction processes like advanced oxidation/reduction (AOP/ARP), which are otherwise promising for degrading soluble PFAS. Furthermore, the low density and small size of many MPs allow them to bypass sedimentation in primary clarifiers, carrying their PFAS cargo into secondary treatment and ultimately into the effluent, thus becoming a pathway for the discharge of both pollutants into receiving waters. As Ma et al. [6] demonstrated, MPs in WWTPs act as both carriers and sources of PFAS, complicating the mass balance and fate modeling essential for effective plant design and operation. In soil remediation, the MP-PFAS complex can similarly subvert standard practices. For example, soil washing or pump-and-treat systems designed to mobilize and capture dissolved PFAS may be ineffective if a significant portion of the PFAS inventory is sequestered on and within MP particles, which may not be mobilized by the same processes.

However, this very challenge points toward a significant opportunity: treatment technologies that effectively capture or destroy MPs can simultaneously remove a substantial vector for PFAS, providing a dual-remediation benefit. The most promising approaches are those that target the destruction of the plastic matrix itself, thereby liberating and subsequently destroying the associated PFAS. Among these, pyrolysis stands out as a particularly viable technology, especially for the management of biosolids, a major sink for both MPs and PFAS in WWTPs. Biosolids are notoriously difficult to treat due to their complex matrix and high concentration of persistent contaminants. Keller et al. [31] conducted a seminal study on the pyrolysis of biosolids at temperatures ranging from 400 to 700 °C. Their results were highly encouraging: pyrolysis resulted in the near-complete elimination of contaminants. They reported that no trace of PFAS was detectable even at 400 °C, and the overall mass removal of PPCPs, including PFAS, was over 99.9%. Concurrently, MP removal via pyrolysis ranged from 91 to 97%. This simultaneous destruction is achieved because the high-temperature, anoxic conditions of pyrolysis break down the organic plastic polymers into syngas and bio-oil, while the thermal energy also cleaves the robust carbon-fluorine bonds in PFAS, leading to defluorination and destruction.

The advantages of pyrolysis extend beyond mere destruction. The process transforms waste into value-added products. The resulting biochar is a stable, carbon-rich material that can be used as a soil amendment or a filtration medium, effectively sequestering carbon and closing the nutrient loop. Keller et al. [31] further highlighted its economic viability, noting that the techno-economic analysis indicates that pyrolysis may generate significant cost savings, and revenue from the sale of biochar, sufficient to more than cover the investment and operating costs. This positions pyrolysis not just as an end-of-pipe treatment, but as a cornerstone of a circular economy strategy for wastewater utilities. For broader water treatment, technologies like membrane filtration (e.g., reverse osmosis, nanofiltration) and advanced coagulation that are effective for MP removal would also co-remove particle-sorbed PFAS, thereby improving overall treatment train efficacy. In conclusion, while the MP-PFAS nexus complicates remediation, it also forces a paradigm shift toward integrated, destructive technologies like pyrolysis, which offer a comprehensive and sustainable solution for managing these intertwined persistent pollutants.

## 4. Combined Toxicity: Unveiling Synergistic Impacts

The true ecological risk of the microplastic–PFAS nexus is most acutely revealed not merely through their co-occurrence, but through their combined toxicological effects on aquatic organisms. Moving beyond individual pollutant studies, recent research demonstrates that co-exposure often leads to unexpected and amplified adverse outcomes, disrupting physiological processes from the molecular to the organismal level. The effects are particularly pronounced in the induction of oxidative stress, genotoxicity, and the disruption of crucial metabolic pathways, with evidence pointing frequently to synergistic interactions that exceed the sum of the individual parts.

### 4.1. Cellular and Molecular Level Effects

At the cellular and molecular level, co-exposure to MPs and PFAS triggers a cascade of stress responses, with enhanced oxidative stress being a central mechanism. The simultaneous physical irritation from MPs and the chemical insult from PFAS can overwhelm an organism’s antioxidant defense systems. This is vividly illustrated in aquatic plants and algae. Zhao et al. [16] conducted a transcriptomic analysis on *Chlorella sorokiniana* exposed to polystyrene MPs (PS-MPs) and PFOA. They found that co-exposure resulted in a greater number of differentially expressed genes (4379) than single exposures, and these genes were predominantly associated with oxidative stress and antioxidant systems. The study revealed that PS-MPs increased PFOA bioavailability by altering cell membrane permeability, and the combined stress primarily regulated the glutathione-ascorbate cycle, a key antioxidant pathway, leading to aggravated cellular damage. This synergy at the transcriptional level explains observations of severe lipid peroxidation and membrane damage that are consistently more severe under co-exposure conditions.

This disruptive synergy extends to fundamental metabolic processes, particularly lipid metabolism. Research on higher organisms reveals that the MP-PFAS complex can disrupt systemic energy homeostasis. Jiang et al. [32] investigated the combined toxicity of PS-MPs and perfluorobutane sulfonate (PFBS) on mice, focusing on the gut-liver axis. Their metabolomics analyses revealed that co-exposure had the most pronounced impact on lipid metabolism disorders, significantly more than either pollutant alone. The mechanism involved a vicious cycle: exposure induced gut microbiota dysbiosis and damaged the gut barrier, which in turn disturbed the enterohepatic circulation of bile acids and other lipid regulators. This disruption along the gut-liver axis led to significant lipid accumulation and inflammation in the liver, demonstrating how the combined pollutant exposure can disrupt intricate inter-organ communication to exacerbate metabolic disease.

**Figure 6:**
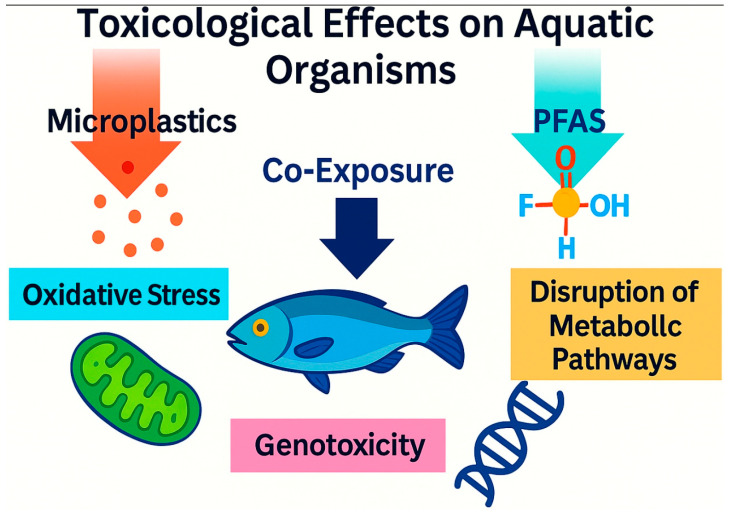
Cellular and molecular level effects of PFAS and microplastics during the co-exposure in aquatic ecosystems.

Furthermore, the nexus inflicts damage at the most fundamental level of genetic integrity and neuronal development. Genotoxicity and neurotoxicity have emerged as critical endpoints of concern. Huang et al. [33], using the regenerative planarian model, showed that PS and PFOS alone caused significant DNA damage, while their combination could either aggravate or attenuate this damage depending on concentration, indicating a complex interactive effect on genomic stability. Perhaps more alarmingly, their study demonstrated that both pollutants, alone and in combination, altered the expression of neuronal genes and impeded the development of the nervous system in regenerating planarians (Figure 6). This provides clear molecular evidence that the MP-PFAS nexus can disrupt neurogenesis, suggesting potential consequences for behavior and neural function in higher organisms. Collectively, these studies from algae to mice confirm that the combined exposure to MPs and PFAS creates a unique chemical stressor that can synergistically attack multiple cellular targets, leading to enhanced oxidative stress, metabolic dysfunction, genotoxicity, and neurotoxicity that far exceed the risks posed by each contaminant in isolation.

### 4.2. Impacts on Key Species and Ecosystems: Primary Producers

As the foundational base of aquatic food webs, primary producers such as algae and submerged plants are critical to ecosystem function, and their vulnerability to the MP-PFAS nexus poses a threat to entire aquatic communities. Exposure to this combined pollution inflicts damage through both physical and chemical pathways, leading to significant inhibition of photosynthesis, stunted growth, and altered metabolic functions. The effects are not merely additive; the presence of microplastics can modulate the toxicity of PFAS, often exacerbating the negative outcomes for these essential organisms.

In algae, the combination of MPs and PFAS creates a multi-faceted stressor. Zhao et al. [12] investigated the response of *Chlorella sorokiniana* to polystyrene microplastics (PS-MPs) and PFOA, identifying distinct yet interacting toxicity mechanisms. They found that PS-MPs primarily inhibited photosynthesis through a shading effect, physically blocking light, and causing direct physical damage to cells. In contrast, PFOA primarily induced oxidative stress by generating reactive oxygen species (ROS). When exposed to both contaminants, these effects were aggravated. The co-exposure led to the highest inhibition rate of algal growth, as the physical barrier created by the MPs compounded the biochemical disruption caused by PFOA. The study further revealed that the algae’s defense mechanisms, such as the secretion of extracellular polymeric substances (EPS) and the activation of the antioxidant system, were activated to a greater degree under co-exposure, indicating a more significant physiological cost to combat the combined stressor. This diversion of energy from growth to defense underscores how the nexus can impair primary productivity at the most fundamental level.

For larger submerged plants, the interactions within the MP-PFAS nexus become even more complex, particularly when factoring in the aging of plastics and the use of emerging PFAS alternatives. Sun et al. [34] exposed the submerged macrophyte *Vallisneria natans* (*V. natans*) to various PFAS (PFOA, PFBA, and GenX) in combination with both pristine and ultraviolet-aged polylactic acid microplastics (PLA and UPLA MPs). Their findings revealed that the aging of MPs is a critical factor that can reverse toxicity outcomes. Moreover, they observed that aged UPLA MPs mitigated the toxicity of PFOA and PFBA but intensified that of GenX. This suggests that the oxidized surface of the aged plastic created different binding sites and interaction potentials with the various PFAS compounds, thereby altering their bioavailability and phytotoxicity. Furthermore, untargeted metabolomics showed that GenX promoted tyrosine metabolism, a key metabolic pathway, but this was suppressed when GenX was co-administered with both types of MPs. This metabolic disruption highlights that the nexus interferes with core biochemical networks in plants. The application of integrated biomarker response (IBR) and partial least squares-structural equation modeling (PLS-SEM) analyses confirmed that the interactions were not uniform; they ranged from antagonistic to synergistic depending on the specific PFAS and the age of the co-occurring MP. This complexity demonstrates that the impact on primary producers is highly context-dependent, influenced by the specific identity and history of the pollutants, making predicting ecological consequences a formidable challenge. In short, the MP-PFAS nexus imposes a significant burden on aquatic primary producers. It disrupts the vital process of photosynthesis through both physical and oxidative mechanisms, forces an energetically costly activation of defense systems, and disrupts core metabolism. The fact that these effects are often synergistic and influenced by the environmental aging of plastics means that the risks to ecosystem foundation and stability are substantially greater than previously assumed from single-pollutant studies.

### 4.3. Invertebrates: Chronic Toxicity and Developmental Failures

**Figure 7:**
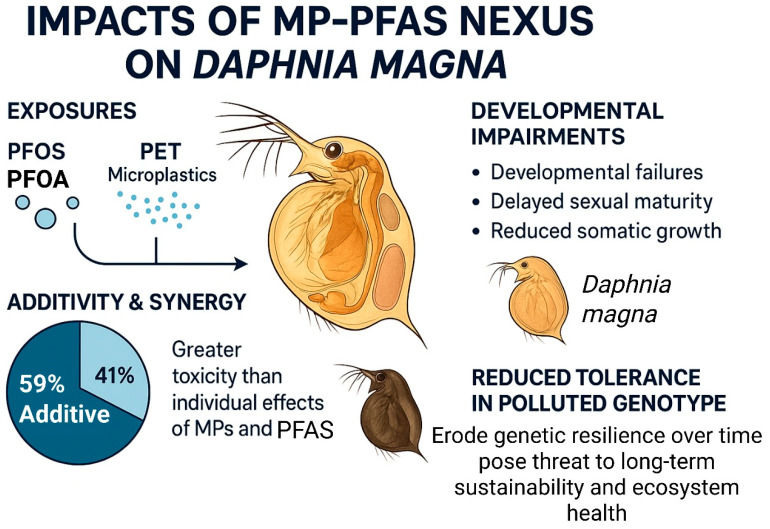
Impact of PFAS-MPs co-exposure on the invertebrate (*Daphnia magna*) leading to the toxicity upon co-exposure in individual and combined pollutants.

The impacts of the MP-PFAS nexus are starkly revealed in chronic toxicity studies using invertebrate sentinel species, which demonstrate that co-exposure can lead to severe developmental impairments and population-level consequences. The water flea *Daphnia magna*, a cornerstone of freshwater ecosystems and ecotoxicological testing, is particularly vulnerable. The research by Soltanighias et al. [15] provides a comprehensive and alarming insight into these effects. Their study investigated the chronic toxicity of PFOS, PFOA, and polyethylene terephthalate microplastics (PET) on two genotypes of *Daphnia magna*. Unlike conventional short-term studies, their life-cycle analysis revealed profound impacts on the organisms’ entire development and reproductive fitness (Figure 7). They found that co-exposure to PFAS and MPs caused developmental failures, delayed sexual maturity, and reduced somatic growth. Crucially, the study demonstrated that the combined effect of these persistent chemicals was 59% additive and 41% synergistic, with no antagonistic interactions observed. This means that in a significant number of cases, the toxicity of the mixture was greater than the sum of the individual effects of MPs and PFAS, leading to unexpectedly severe outcomes. Furthermore, the study highlighted a critical ecological insight: a genotype with a historical exposure to environmental pollution showed reduced tolerance to the MP-PFAS mixture, suggesting that the cumulative fitness costs from past pollution have eroded the population’s capacity to cope with new, combined stressors. This indicates that the nexus does not only cause direct mortality but can also erode genetic resilience over time, posing a long-term threat to population stability and ecosystem health.

### 4.4. Microbiome: Disruption of Essential Rhizosphere Communities

The detrimental effects of the MP-PFAS nexus extend beyond macroscopic organisms to the foundational level of aquatic ecosystems: the microbial communities that drive biogeochemical cycling and support plant health. Research on the rhizosphere, the dynamic interface between plant roots and soil, reveals that this combined pollution can significantly disrupt the structure and function of these essential bacterial consortia. Chen and Hua [28] investigated the eco-environmental responses of the rhizobacteria community associated with the floating macrophyte *Eichhornia crassipes* (water hyacinth) to co-stress from PFAS and polystyrene microplastics (PS-MPs). Their high-throughput sequencing analysis demonstrated that exposure to these pollutants significantly altered the richness and diversity of the bacterial community. Notably, they found that PS-MPs enhanced the deterministic assembly processes driven by certain PFAS (F-53B and GenX), meaning the pollutants acted as a strong environmental filter, selectively eliminating sensitive microbial taxa and reducing the stochastic, random distribution of species that often promotes diversity. This led to a significant narrowing of the ecological niche breadths for the rhizobacteria, indicating a loss of functional specialization and resilience. Furthermore, the study connected these structural shifts to impaired ecosystem services, showing that the contaminants decreased the functional potential of the microbiome for anaerobic processes and ammonia nitrogen utilization. This disruption of key nutrient-cycling pathways underscores a critical threat: the MP-PFAS nexus can degrade the health of plant-microbe systems not merely by direct toxicity, but by dismantling the vital microbial partnerships that underpin nutrient acquisition and overall ecosystem productivity, thereby causing a cascade of functional degradation throughout the habitat.

### 4.5. Toxicity in Vertebrates: From Aquatic Organisms to Mammalian Models

While impacts on lower trophic levels are crucial, the effects of the MP-PFAS nexus on vertebrates raise significant concerns for ecosystem health and potential human implications. Evidence from fish and mammalian models indicates that co-exposure can lead to synergistic toxicity, disrupting key physiological systems. In aquatic vertebrates, particularly fish, the combined exposure manifests as enhanced metabolic and oxidative stress. The presence of MPs can exacerbate the well-documented toxicity of PFAS, such as liver damage and endocrine disruption. Co-exposure studies often report higher inflammatory responses and greater accumulation of PFAS in tissues like the liver and gills compared to PFAS exposure alone, likely due to the MP vector facilitating gill uptake and dietary ingestion. This can lead to impaired growth, reduced survival rates, and abnormal swimming behavior, indicating neurotoxic effects.

The interaction becomes particularly complex in mammalian models, which provide insights into potential human health risks. Jiang et al. [32] demonstrated that the co-exposure of PS-MPs and PFBS in mice led to a synergistic disruption of the gut-liver axis. This was characterized by gut microbiota dysbiosis, damage to the intestinal barrier, and subsequent dysregulation of hepatic lipid metabolism, culminating in pronounced liver steatosis (fatty liver disease). The MPs were found to enhance the bioavailability and tissue retention of PFBS, creating a more severe pathological outcome than either contaminant could produce independently. This suggests the nexus can amplify metabolic syndrome-like conditions. Furthermore, research indicates toxicity beyond metabolic organs. Studies report that co-exposure can induce higher levels of genotoxicity in lymphocytes and more significant oxidative damage in the brain and renal tissues of vertebrate models. The combination also appears to aggravate the immunotoxicity of PFAS, leading to a greater suppression of immune function. These findings across multiple vertebrate systems underscore that the MP-PFAS nexus is not merely an ecological issue but a potential driver of systemic health failures in higher organisms, highlighting an urgent need for more targeted toxicological assessment in vertebrates.

## 5. Emerging Frontiers and Future Perspectives

### 5.1. The Climate Change Connection: An Amplifying Feedback Loop

The pervasive challenge of the MP-PFAS nexus is set within the broader context of a changing global climate, which acts as a threat multiplier, amplifying both the release and the ultimate impact of these pollutants. As articulated by Gander [3], climate change is not a separate environmental issue but a force that intensifies the life cycle of the nexus. The increasing frequency and intensity of extreme weather events, such as storms and floods, exacerbate the mobilization and redistribution of both microplastics and PFAS from terrestrial sources into aquatic ecosystems. Heavy rainfall can wash larger volumes of contaminated stormwater from urban and industrial areas into rivers and lakes, while also scouring landfills and contaminated sites, thereby releasing historically stored pollutants in pulses. Furthermore, climate change can directly influence the degradation and release of these contaminants. Rising global temperatures can accelerate the physical weathering of plastic debris, increasing the generation rate of secondary microplastics. Crucially, Gander [3] also highlights a particularly concerning feedback loop: the breakdown of microplastics is itself a source of greenhouse gases such as methane and ethylene, which in turn contribute to further climate change. This creates a vicious cycle whereby a warming climate promotes the fragmentation of plastics, which releases more greenhouse gases and simultaneously produces more MPs that can vector PFAS. This interconnected relationship means that strategies to mitigate climate change and manage plastic pollution are inextricably linked, and failure to address one will inevitably exacerbate the risks posed by the other.

### 5.2. Human Health Implications: From Aquatic Ecosystems to Human Exposure

The environmental MP-PFAS nexus represents a significant and direct threat to human health, primarily through the ingestion of contaminated food and water. The potential for this complex to enter the human body creates an exposure pathway that can lead to a higher internal dose of PFAS than from water or food alone. Seafood consumption is a major route, as the complex can efficiently travel up the aquatic food web. Yu et al. [30] provided compelling field evidence for this, finding that marine fish with a higher burden of microplastics also had significantly higher concentrations of perfluoroalkyl acids. Their structural equation modeling confirmed that the microplastics ingested by the fish contributed directly to the level of chemical contamination in their tissues. This trojan horse effect means that consuming seafood introduces not just PFAS, but also the MP vectors that may continue to desorb PFAS in the human gut, potentially enhancing bioavailability and absorption.

Beyond seafood, drinking water is a critical exposure route. Du et al. [35] used molecular dynamics simulations to investigate this, revealing that hydrophobic PFAS are more easily adsorbed by MPs in drinking water. They also highlighted a concerning interaction, finding that 59.38% of plastic additive combinations had synergistic toxicity effects with PFAS on female reproductive health. This suggests that the combined pollutant ingested via water could pose a greater risk than the individual chemicals. Perhaps most alarmingly, exposure is not limited to environmental contamination; it can occur directly from food contact materials. Cole et al. [36] demonstrated that microplastics and PTFE (a fluoropolymer and source of PFAS) can be released from cookware into food during preparation. Their study estimated that using plastic cookware could introduce thousands of microplastics into a single meal, simultaneously leaching PTFE particles and associated PFAS. This direct contamination of food creates a pervasive exposure route, implicating the MP-PFAS nexus as a potential contributor to the body burden of these chemicals in the general population and raising concerns about associated health risks, including metabolic, developmental, and reproductive effects.

### 5.3. Novel Analytical and Modeling Approaches: Deconstructing the Nexus

Advancements in analytical chemistry and computational modeling are providing unprecedented tools to deconstruct the complexity of the MP-PFAS nexus, moving from simple observational studies to mechanistic and predictive understandings. In the analytical realm, techniques that can simultaneously characterize the plastic polymer and associated contaminants are groundbreaking. Brunnbauer et al. [37] demonstrated the power of a simultaneous Laser-Induced Breakdown Spectroscopy and Laser Ablation-Inductively Coupled Plasma-Mass Spectrometry (LIBS/LA-ICP-MS) setup. This approach allows for the correlated chemical imaging of a single particle: LIBS rapidly identifies the polymer type (e.g., by detecting fluorine for PTFE) and LA-ICP-MS provides ultra-sensitive, quantitative data on associated metals. This is particularly powerful for identifying fluorinated polymers, which are both a type of MP and a direct source of PFAS, enabling researchers to directly visualize and quantify this specific link within environmental samples.

Complementing these analytical advances, in silico (computational) modeling offers a way to predict interactions and toxicity at a molecular level, saving time and resources. Linear Solvation Energy Relationship (LSER) models, as developed by Hatinoglu et al. [38], are a prime example. They created the first LSER model for PFAS adsorption onto MPs, systematically accounting for the ionization state of the pollutants. Their model revealed that the polarizability and hydrophobicity of anionic PFCA are the most significant contributors to their adsorption, while van der Waals interactions with water decrease binding affinity. This provides a quantitative framework to predict the behavior of untested PFAS-MP combinations. At an even more fundamental level, Grand Canonical Monte Carlo and Molecular Dynamics (GCMC-MD) simulations, as employed by Enyoh et al. [39], allow us to peer into the nanoscale interactions. Their simulations delineated the role of the fluoroalkyl tail in binding and quantified the contribution of different interaction energies (e.g., van der Waals, electrostatic), providing atomic-level insight that is impossible to obtain experimentally. Together, these sophisticated techniques are transforming our ability to predict the formation, stability, and ultimate risk of the countless potential MP-PFAS combinations in the environment.

A significant hurdle in advancing the science of the MP-PFAS nexus lies in the formidable analytical challenges and the resulting methodological variability across studies. The accurate quantification of PFAS, especially when sorbed to a complex MP matrix, is non-trivial. The first challenge is the extraction efficiency. There is no standardized method for simultaneously extracting a wide range of PFAS (from short-chain to long-chain, and from precursors to PFAAs) from different polymer types. The use of solvents like methanol may efficiently extract some PFAAs but can be less effective for more hydrophobic precursors or for PFAS that have undergone strong, specific interactions with weathered MP surfaces.

Secondly, the analysis itself is plagued by issues such as adsorption to laboratory materials, background contamination from PTFE-containing labware, and ion suppression/enhancement in LC-MS/MS analysis when dealing with the complex MP leachates or digestates. This is compounded when attempting to analyze the vast number of unknown PFAS (the dark matter of PFAS) for which analytical standards are unavailable. As Brunnbauer et al. [37] demonstrated, novel techniques like simultaneous LIBS/LA-ICP-MS are powerful for polymer identification and elemental analysis, but they currently lack the sensitivity for trace-level PFAS quantification on individual particles.

This methodological variability has critical implications. It leads to difficulties in comparing adsorption coefficients (Kd values) across different studies, as results are highly dependent on the experimental conditions (MP/water ratio, salinity, contact time) and analytical protocols used. Salawu et al. [14] reported that PFOS desorption exceeded 120% underscores how traditional methods can fail to account for the inherent PFAS content within virgin MPs. This calls for the development of standardized reference materials and harmonized analytical protocols specifically designed for MP-sorbed contaminants. Without such standards, reconciling data and building robust, predictive models for the environmental behavior of the MP-PFAS nexus will remain a profound challenge.

### 5.4. Policy and Regulatory Gaps: The Urgent Need for a Holistic Framework

**Figure 8:**
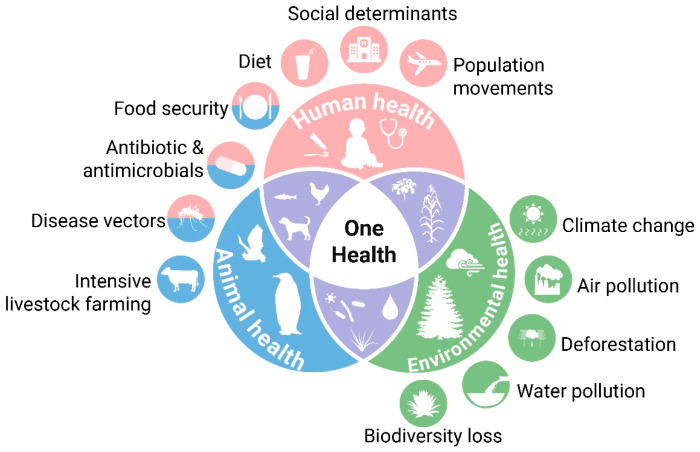
A one-health approach to protect environmental ecosystems from the exposure of microplastics and PFAS.

The established paradigm for chemical risk assessment, which largely evaluates contaminants in isolation, is fundamentally inadequate for addressing the complex and synergistic threats posed by the MP-PFAS nexus. This regulatory gap creates a false sense of security and perpetuates the unchecked release of these pollutants. As Belmaker et al. [40] compellingly argue in their analysis of the Israeli context, the current fragmented approach, which often regulates single substances or specific product categories, fails to capture the reality of combined exposure and its amplified health effects. The authors highlight the pressing need for research into the ecological impacts of MPs on soil health and the importance of mitigation strategies aimed at reducing contamination and exposure risks, especially concerning PFAS. This call to action underscores that our regulatory frameworks are lagging behind the scientific understanding of co-contaminant behavior. To protect environmental and public health effectively, risk assessment models must be updated to incorporate mixture toxicity and vector-mediated exposure. This means that the safety of a chemical like PFAS can no longer be assessed without considering the ubiquity of microplastics that can concentrate it and alter its bioavailability. Furthermore, regulations must move beyond a narrow focus on end-of-pipe solutions and embrace a life-cycle approach that addresses the entire trajectory of these pollutants, from their intentional use as additives in plastic manufacturing to their ultimate breakdown and interaction in the environment (Figure 8). Closing this policy gap is not merely an academic exercise; it is an urgent necessity to proactively manage the cocktail effect of multiple pollutants and to develop legislation that is as interconnected and sophisticated as the environmental challenges we now face.

## 6. Conclusions

The body of evidence synthesized in this review unequivocally demonstrates that microplastics and per- and polyfluoroalkyl substances (PFAS) are not merely co-occurring pollutants but are engaged in a dynamic and multifaceted relationship, the MP-PFAS Nexus. This nexus is characterized by a complex interplay of mechanisms where microplastics act as pervasive vectors, concentrators, and even secondary sources for PFAS. The interaction is not static; it is continuously modulated by environmental aging processes like UV degradation and biofilm formation and is exquisitely sensitive to water chemistry parameters such as pH and ionic strength. The most critical finding that emerges is that the combined ecological impact of this nexus is often synergistic, leading to enhanced toxicity, developmental failures, and metabolic disruptions across all levels of biological organization, from primary producers like algae to sentinel invertebrates and fish. This synergy challenges the very foundation of traditional risk assessment, which evaluates contaminants in isolation.

The environmental implications are profound. The formation of MP-PFAS complexes alters the fate and transport of both contaminants, facilitating the long-range distribution of PFAS and promoting their accumulation in benthic sediments and their transfer through aquatic food webs. This Trojan Horse effect not only increases the body burden in aquatic organisms but also poses a direct and escalating threat to human health through the consumption of contaminated seafood and drinking water. The interconnected nature of this problem is further exacerbated by broader environmental challenges, most notably climate change, which acts as a threat multiplier by increasing the mobilization of both pollutants and creating a feedback loop through the release of greenhouse gases from degrading plastics. Moving forward, a paradigm shift is urgently required in how we monitor, assess, and mitigate these persistent pollutants. First, regulatory frameworks must evolve to account for mixture toxicity and vector-mediated exposure. The current approach of regulating single substances is obsolete in the face of such complex interactions. Second, research must prioritize the development and implementation of advanced destructive technologies, such as pyrolysis for biosolids, which offer a promising pathway to simultaneously eliminate both MPs and PFAS, thereby closing the loop in a circular economy model. Finally, global policies must embrace a life-cycle approach, targeting the production of plastic and PFAS at the source and preventing the creation of this problematic nexus altogether. In short, viewing MPs and PFAS through the lens of an interconnected nexus is not merely an academic exercise; it is an essential step toward a more accurate and holistic understanding of modern environmental contamination. Addressing this intertwined threat demands integrated science, innovative technology, and courageous policy that together can safeguard ecosystem integrity and public health for future generations.

## Data Availability

No new data were created or analyzed in this study. Data sharing is not applicable to this article.
